# Spreading rates of bacterial colonies depend on substrate stiffness and permeability

**DOI:** 10.1093/pnasnexus/pgac025

**Published:** 2022-04-15

**Authors:** Merrill E Asp, Minh-Tri Ho Thanh, Danielle A Germann, Robert J Carroll, Alana Franceski, Roy D Welch, Arvind Gopinath, Alison E Patteson

**Affiliations:** Physics Department, Syracuse University, Syracuse, NY 13244, USA; BioInspired Institute, Syracuse University, Syracuse, NY 13244, USA; Physics Department, Syracuse University, Syracuse, NY 13244, USA; BioInspired Institute, Syracuse University, Syracuse, NY 13244, USA; Physics Department, Syracuse University, Syracuse, NY 13244, USA; BioInspired Institute, Syracuse University, Syracuse, NY 13244, USA; Physics Department, Syracuse University, Syracuse, NY 13244, USA; BioInspired Institute, Syracuse University, Syracuse, NY 13244, USA; BioInspired Institute, Syracuse University, Syracuse, NY 13244, USA; Biology Department, Syracuse University, Syracuse, NY 13244, USA; BioInspired Institute, Syracuse University, Syracuse, NY 13244, USA; Biology Department, Syracuse University, Syracuse, NY 13244, USA; Department of Bioengineering, University of California, Merced, Merced, CA 95343, USA; Health Sciences Research Institute, University of California, Merced, Merced, CA 95343, USA; Physics Department, Syracuse University, Syracuse, NY 13244, USA; BioInspired Institute, Syracuse University, Syracuse, NY 13244, USA

**Keywords:** bacterial colonies, biofilms, hydrogels, mechanobiology, traction force microscopy

## Abstract

The ability of bacteria to colonize and grow on different surfaces is an essential process for biofilm development. Here, we report the use of synthetic hydrogels with tunable stiffness and porosity to assess physical effects of the substrate on biofilm development. Using time-lapse microscopy to track the growth of expanding *Serratia marcescens* colonies, we find that biofilm colony growth can increase with increasing substrate stiffness, unlike what is found on traditional agar substrates. Using traction force microscopy-based techniques, we find that biofilms exert transient stresses correlated over length scales much larger than a single bacterium, and that the magnitude of these forces also increases with increasing substrate stiffness. Our results are consistent with a model of biofilm development in which the interplay between osmotic pressure arising from the biofilm and the poroelastic response of the underlying substrate controls biofilm growth and morphology.

Significance Statement Many bacteria can transition between individual swimming behavior and multicellular biofilm communities in response to changes in their environment. First contact with a surface is an important cue for bacteria to begin transitioning into biofilm colonies. However, the mechanisms by which bacteria sense and respond to surfaces is poorly understood. Here, we show how the collective expansion of biofilm colonies can be modulated by changing physical properties of the growth substrate, such as substrate stiffness and porosity. By developing tunable synthetic hydrogel substrates, we show that increasing substrate stiffness can enhance biofilm expansion independently of changes in substrate porosity. Our results point to a hitherto-unrecognized mode of collective surface spreading in the prokaryote kingdom.

## Introduction

Biofilm formation is an important process in the bacterial lifecycle. Biofilms are multicellular communities of bacteria commonly attached to an external surface (1, 2). Emerging evidence indicates that bacteria sense and respond to variations in the mechanical properties of the surrounding environment, resulting in changes to cell physiology and biofilm morphology (3*–*6). When a bacterium makes contact with a surface, it initiates a program of gene expression that promotes colonization and secretion of extracellular polymeric substances (EPS) that self-encapsulate the cells and gives the biofilm its structure (7, 8). The biofilm thus consists of both cells and EPS components, growing as a result of both cell division and EPS deposition (9). Colony growth is aided by the production of surfactants (9) and EPS-generated osmotic pressure gradients, which facilitate nutrient uptake from the substrate (10, 11). Thus, the physical properties of the underlying substrate have the potential to disrupt structural and functional aspects of cell attachment and function that contribute to biofilm phenotypes.

The vast majority of biofilm experiments are conducted on the surface of an agar gel. Agar was introduced in 1882 by Angelina Fanny Hesse and gained popularity through Robert Koch (12) because it is inert to bacteria degradation. However, agar is isolated from marine algae and is an undefined media, as its chemical composition is not entirely known (13). Agar variability from the isolation process makes it difficult to define and reproduce its chemical and physical properties (14, 15).

A common feature of these studies is that biofilm expansion decreases with increasing agar concentration (5, 11, 16, 17)_._ Increasing agar concentration increases agar network stiffness but also impacts other properties of the gel, such as the hydrogel pore size (11). Agar is typically prepared in the range 0.5%–2% agar in a nutrient-rich media, forming a hydrogel comprised of a porous solid network and the nutrient-rich interstitial fluid that permeates through the network. On stiff agar, the pore size is smaller and the rate of nutrient transport through the substrate and to the biofilm decreases (11, 18). A number of studies have attributed this inhibited biofilm growth on stiff agar to lack of nutrients rather than stiffness per se (11, 18). On the other hand, there are studies indicating substrate stiffness can separately modify biofilm shape and expansion by mediating adhesion (19, 20) and frictional forces between the biofilm and the substrate (21). The extent to which biofilm growth depends on the combined effects of substrate stiffness and nutrient availability is thus an open question, and current bacteria culture substrates largely cannot separate the effects of these two properties on biofilm growth.

Here, we report the development of polyacrylamide (PAA) hydrogels with tunable matrix stiffness and matrix porosity to determine their integrated effects on biofilm growth. We identify a new regime in the limit of purely elastic substrates in which bacteria colonies spread out faster on stiffer substrates compared to softer ones, which is opposite of conventional agar. Our study focuses on the bacterium *Serratia marcescens*, which is a common model organism for collective motion and behavior (6, 22*–*24), but we also show that *Pseudomonas aeruginosa*, *Proteus mirabilis*, and *Myxococcus xanthus* expand faster on stiffer substrates than soft ones. A major advantage of PAA gels is that unlike agar they linearly deform in response to a wide range of stress, which enables facile force calculations. Using traction force microscopy (TFM)-based techniques, we show that bacteria colonies generate transient forces that are correlated over length scales much larger than a single bacterium, and that the magnitude of these forces increases with increasing substrate stiffness. Our results are consistent with a model in which biofilm development is impacted by osmotic pressure gradients between the biofilm and the substrate and the substrate's poroelastic response.

## Results

### Design and characterization of PAA hydrogels

**Fig. 1.**
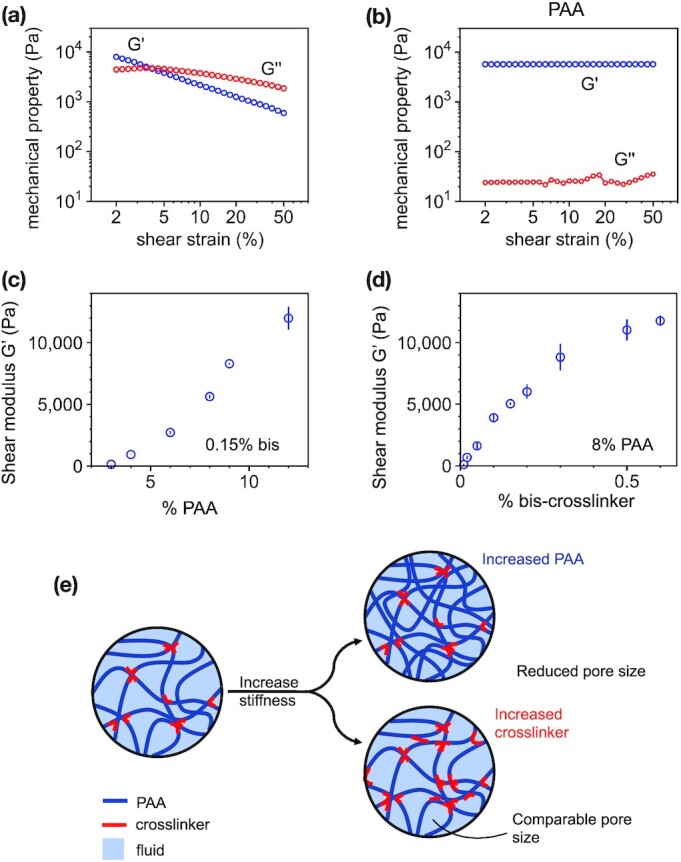
Substrate characterization using a stress-controlled rheometer. The shear storage modulus G’ and loss modulus G’’ as a function of shear strain for both (a) agar and (b) PAA gels. PAA gels are prepared by either (c) increasing PAA concentration or (d) chemical crosslinker bis-acrylamide. (e) Schematic representation illustrating the effects of increasing PAA polymer concentration vs. chemical crosslinker on fluid permeability in the network. Error bars denote standard error from 3 + independent trials per condition.

In this study, we used both PAA gels and conventional agar as a point of comparison. To characterize the mechanical properties of the gels, we measured under shear their elastic storage modulus G′, which quantifies their resistance to shear deformations, and their viscous loss modulus G′′, which quantifies viscous energy dissipation, with an oscillatory rheometer. As shown in Fig. 1(a), agar exhibits nonlinear shear softening; its shear modulus decreases from approximately 10–1 kPa as shear strain rises from 2% to 50%. The mechanical response of PAA to shear strain differs from agar. As shown in Fig. 1(b), PAA gels form linearly elastic gels, with near constant G’ over the applied strain range.

The shear modulus G’ sets the extent to which a material deforms under an applied shear stress. The nonlinear shear softening is a property of complex materials and demonstrates that agar is softer when probed at higher deformations compared to small ones. If biofilms deform their substrate at magnitudes that vary over time or under different experimental conditions, then the shear modulus of the agar substrate will vary in response to the applied deformation and the biofilm would experience a different mechanical resistance from the substrate.

Agar also exhibits significant viscoelasticity, with a viscous loss modulus G’’ of 10%–50% of the storage modulus G’ at least for small strain values (2%–5%) at a frequency of 1 Hz. This data suggests that as a substrate for biofilm growth, agar dissipates energy and relaxes applied stresses that might be relevant to outward growth of the colony. PAA gels, in contrast, exhibit negligible viscous dissipation, consistent with prior work (25*–*27).

Unlike agar and most other bacterial growth substrates, the shear (elastic) moduli of PAA gels can be tuned by either crosslinker concentration or polymer concentration, which allows tunable control of matrix stiffness and matrix pore size (28). In order to distinguish between the effects of substrate stiffness and substrate permeability on biofilm growth, we thus designed PAA gels (Table S1, Supplementary Material) with shear moduli G’ ranging from 100 to 10,000 Pa by varying either the amount of acrylamide or the amount of the chemical crosslinker bis (Fig. 1c and d). These two parameters, acrylamide concentration and crosslinker concentration, have two different effects on network permeability (Fig. 1e) (28). Increasing the concentration of acrylamide monomer results in a denser, stiffer PAA network with a smaller pore size, and thus lower permeability. Increasing the concentration of crosslinker links together the same density of PAA polymers at a greater number of sites, increasing the network stiffness without significantly changing pore size. These effects are illustrated schematically in Fig. 1(e). We confirmed these expectations using effective diffusion and force indentation experiments to estimate the effective pore sizes of the PAA gels (Table 1; Figures S1 and S2, Supplementary Material). Here, we note here that effective transport of nutrients through the network depends on both molecular diffusion and poroelastic transport of solvent as gels swell or deswell, causing fluid fluxes. We refer to nutrient transport and diffusivity in terms of effective diffusivities that combine the effects of both. The effectivity diffusivities measured here range from approximately 70 to 175 μm ^2^/second, which is consistent with prior literature values for PAA gels (29) accounting for differences in the shear modulus G’ of the gels (30).

**Table 1.** Effective pore size measurements of PAA gels (details in the Supplementary materials).

% PAA	% Bis	Effective diffusivity (μm^2^/second)	Pore size (nm)
3	0.15	170 ± 22	22 ± 5.6
12	0.15	80 ± 20	0.85 ± 0.1
8	0.085	75 ± 10	1.5 ± 0.1
8	0.45	70 ± 10	0.9 ± 0.2

### Substrate stiffness increases biofilm expansion rates

**Fig. 2.**
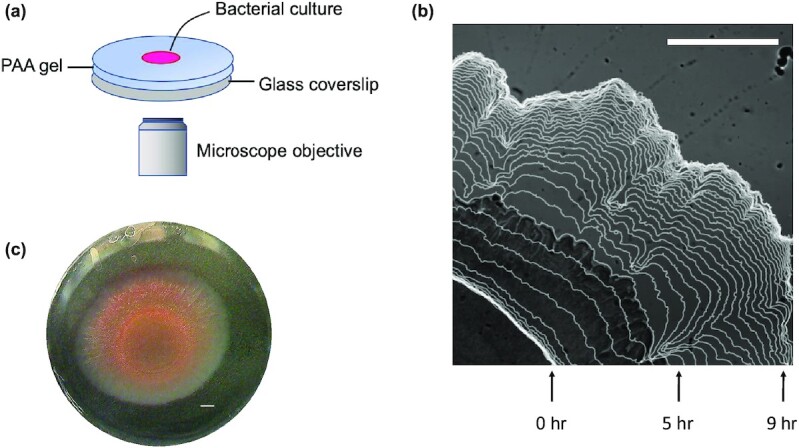
(a) Schematic diagram of the experimental setup. Aliquots of *S. marcescens* were placed on PAA substrates that were 0.8 mm in height. The substrates were maintained at 37°C in a humid stage top incubator. (b) Visualization of the growing biofilm boundary overlaid on a sample image of the biofilm. Images were acquired at 10-minute increments, and boundaries shown here are displayed at 20-minute increments. (c) Color image of full bacterial colony after 15 hours of growth. Scale bars, 1 mm.

Our experimental protocol consists of directly observing the growth of *S. marcescens* colonies on the surface of hydrogel substrates with time-lapse microscopy (Methods). Before inoculation, the PAA gels are soaked multiple times in LB nutrient-rich broth. We deposit a small inoculum of bacteria on the gel surfaces and track x–y positions of the resulting biofilm boundary as it expands over 15-hour time periods, relevant to prior literature reports (21, 31) (Fig. 2). The biofilm boundary is tracked by a custom semiautomated Python script we developed for these videos (Methods; Figure S3, Supplementary Material; and Video S1).

**Fig. 3.**
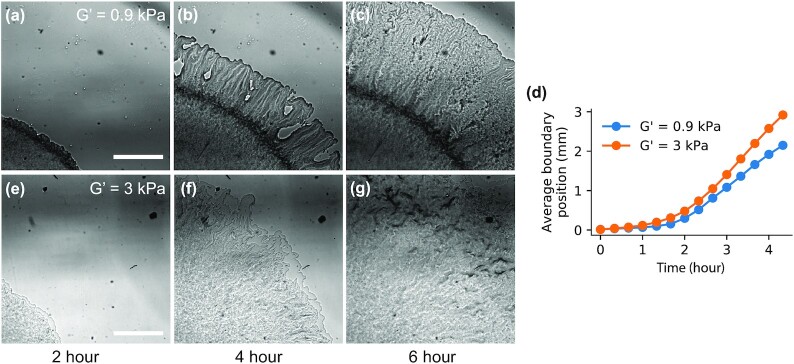
Representative bright field images of *S. marcescens* colonies growing across a soft (a)–(c) and stiff (e)–(g) PAA gel. Variations in gray intensity correlate with the amount of transmitted light through the biofilm, which depends upon both colony density and height. Both the biofilm structure and expansion rate (d) depend on substrate stiffness. Scale bar, 1 mm. Soft gel: G’ = 0.9 kPa, 4% PAA, and 0.15% bis-crosslinker. Stiff gel: G’ = 3 kPa, 6% PAA, and 0.15% bis-crosslinker.

Representative biofilm time-lapse images on a soft (G’ = 0.9 kPa) and a stiff (G’ = 3 kPa) PAA gel are shown in Fig. 3, with videos available as supplementary materials (Videos S2 and S3, respectively). There are notable differences in colony morphology and the collective cell migration speed between the two gel types. While the biofilm surface expansion speed encompasses the collective effects of rates of EPS production, cell division, and cell surface motility, the colony expansion rate is faster on the stiffer PAA gel compared to the softer one, opposite of the behavior on conventional agar substrates.

A central feature of biofilm formation is the production of EPS, which adheres cells to each other and external surfaces. EPS production allows for vertical growth of the colony (32), and also mediates osmotic spreading of the colony edge (10, 18). To determine whether the bacteria colonies were producing EPS, we stained the colonies with a fluorescent biofilm matrix stain and found EPS deposition throughout the colony (Figure S4, Supplementary Material). We also observed wrinkles and surface corrugations on the colony surface, characteristic of EPS production and biofilm formation. To visualize the 3D colony structure, we used a white-light interferometer to map the 3D colony verticalization (Figure S5, Supplementary Material). The colonies on soft substrates were more vertical than colonies on stiff substrates, with colony heights of approximately 25 μm on soft substrates compared to 5 μm on stiff substrates. Compared to 3D imaging methods such as confocal microscopy or white light interferometry, wide-field imaging of the colonies can be gathered in larger numbers with an automated multipoint microscope. Thus, here, we focus on the 2D colony expansion rates as a high throughput metric to screen the effects of substrate stiffness on colony surface dispersal.

**Fig. 4.**
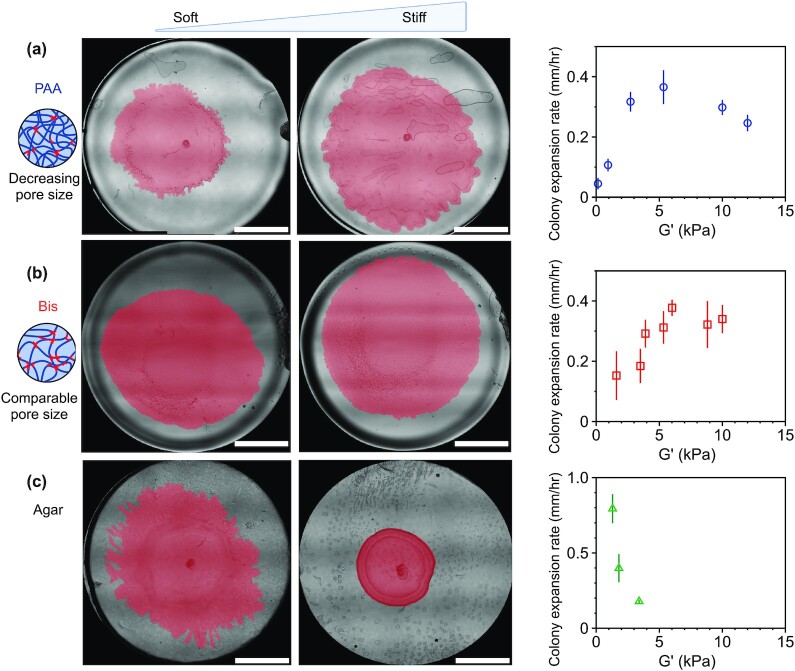
Colony expansion rates depend on substrate composition. Expansion data was collected for (a) PAA gels of varying PAA, (b) PAA gels of varying bis, and (c) agar gels of increasing agar concentrations. While colony size decreases on stiffer agar substrates, the opposite occurs on purely elastic PAA substrates. The figure shows representative colony pictures of *S. marcescens* grown on soft and stiff gels for each gel composition type. The biofilms are manually traced and pseudo-colored pink to enhance imaging contrast. Scale bar, 5 mm. The schematics show the relative effects of varying PAA vs. bis-crosslinker on the network pore size. Biofilm expansion velocities are shown as a function of substrate stiffness (G’) for *S. marcescens* colonies grown on each substrate type (2 hours postinoculation). Data points represent mean of *N* = 3–6 independent colonies for each condition. Error bars denote SEM.

This phenomenon is highlighted in Fig. 4, which shows snapshots of whole biofilm colonies on PAA gels and agar substrates taken several hours after inoculation. We note that the reduced biofilm growth on stiff agar substrates compared to soft agar is a common feature of many different bacteria species, such as *Vibrio cholerae*, *P. mirabilis, M. xanthus*, and*Salmonella enterica* (5, 11, 16, 17). This inhibited biofilm growth on agar has been attributed to the reduction in substrate permeability found in more concentrated agar substrates, which limits the transport of fluid and nutrients from the substrate into the biofilm (11). Another physical factor contributing to biofilm expansion rates is the surface tension between the biofilm and the surface, where decreasing surface tension increases biofilm expansion, by allowing the leading edge to propagate and advance faster (33). Therefore, we measured the surface tension between a fluid droplet and the hydrogel surfaces with a contact angle goniometer (Table S2, Supplementary Material). We found that the contact angle increased from 15° ± 2° to 23.5° ± 1° for 4% and 8% PAA, respectively, suggesting that the effects of surface tension would lead to increased biofilm expansion on softer PAA gels. The strong increase in biofilm expansion on more concentrated PAA gels is thus unexpected from the effects of the hydrogel itself on surface tension (Figs 3 and 4). We note that the colony expansion rates here are significantly slower than swarming expansion rates and no vortical collective flows are observed, which indicate that the colony is in a biofilm state in contrast to a swarming state (18, 34). Cell motility, however, likely does contribute to the expansion process, as a subfraction of cells in a biofilm maintain a motile state ( 35).

To quantify the above observations, we calculate the initial biofilm expansion rates by calculating the boundary velocities from the tracking data. The biofilm velocity is defined here as the average radial displacement of the biofilm boundary over a time interval of Δ*t* = 20 minutes. Here, we use the biofilm boundary velocity as a metric of wild-type collective expansion, not a direct measure of single cell surface motility or bacteria doubling time.

Figure 4 shows the initial colony expansion velocities on PAA and agar substrates when radial expansion of the biofilms are first beginning to be observed, approximately 2 hours postinoculation. Figure 4(a) shows the surface expansion rate for PAA hydrogels of increasing PAA concentration. We find that there is a significant increase in colony expansion rate with increasing substrate stiffness, particularly for substrate stiffness G’ < 5 kPa. For substrate stiffness greater than 5 kPa, the colony velocity seems to saturate with substrate stiffness and then begins to slowly decline on PAA gels (Fig. 4a). These results are strikingly similar for PAA gels with the same range of substrate stiffness, but prepared by increasing bis-crosslinker (Fig. 4b). We note that Fig. 4(b) serves as a control. Unlike increases in PAA, increasing the bis-crosslinker does not significantly modify the network pore size, indicating a distinct effect of substrate stiffness on colony expansion. These results are entirely different from the biofilm growth on agar substrates: the colony velocity decreases dramatically with increased agar concentration (Fig. 4c), even for substrate stiffness less than 5 kPa.

We note that we do observe differences between hydrogels prepared by varying PAA and varying bis of the same hydrogel stiffness G’. These differences are most evident for soft gels, where G’ < 5 kPa (Fig. 4a and b): colonies on the varying PAA gels expand more quickly than colonies on the varying bis gels (*P* < 0.01 for G’ ≅ 3 kPa). In this regime, the varying PAA gels have larger pore sizes (22 nm) than the varying bis gels (1.5 nm; Table 1; Table S3, Supplementary Material). This result is thus consistent with the idea that larger network pore sizes increase colony expansion rate, allowing more nutrient-rich fluid to flow from the substrate into the colony. For G’ < 5 kPa, the expansion rates saturate to approximately the same magnitude, 0.3 mm/hour, for each case.

**Fig. 5.**
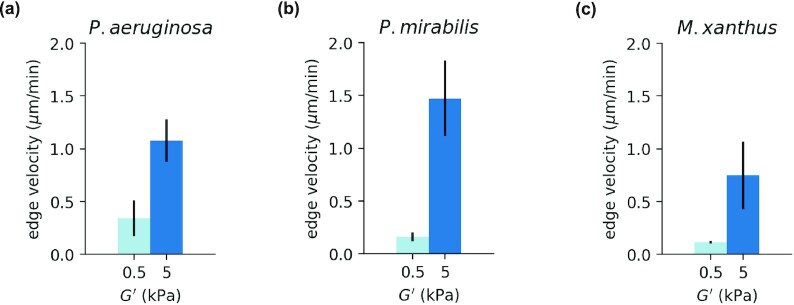
Biofilm expansion rates on soft (G’ = 0.5 kPa) and stiff (G’ = 5 kPa) PAA gels for (a) *P. aeruginosa*, (b) *P. mirabilis*, and (c) *M. xanthus* bacteria species. Data from *N* = 3 colonies. Error bars denote SEM.

Taking into account the diverse bacterial strains that colonize agar, we selected 3 additional bacterial species to test on PAA gels: *P. aeruginosa*, *P. mirabilis*, and *M. xanthus* (Methods). Given the strong effect of substrate stiffness on *S. marcescens* surface expansion (Fig. 4), we selected a soft (G’ ≅ 0.5 kPa) and stiff (G’ ≅ 5 kPa) PAA gel to culture these 3 species (Fig. 5). In each case, we found that biofilm expansion was faster on stiffer PAA than softer PAA (Methods). *Pseudomonas aeruginosa* and *P. mirabilis* are both Gram-negative bacteria known to cause disease in humans. Here, we use *P. aeruginosa* Xen05, which is derived from a human septicemia isolate, and *P. mirabilis* BB2000. *Proteus mirabilis* are well-known for their ability to swarm, a flagella-based rapid surface motility mode, which they are capable of doing over a striking range of surfaces (36). *Myxococcus xanthus*, a member of the δ-Proteobacteria, displays a wide range of multicellular emergent behaviors (37, 38). *Myxococcus xanthus* have two well-characterized motility modes, social (S)-motility powered by Type IV pili (39) and adventurous (A)-motility, powered by an inner membrane motor that applies force to the substrate at adhesions (40, 41); they do not have flagella. While *M. xanthus* is well-known for its display of dynamic fruiting body formation when starved (37, 38), here we focus on its collective biofilm expansion in growth media. Given the different motility modes of three different bacteria species, our results suggest that for PAA hydrogels increasing biofilm expansion rates on substrates with increasing stiffness is a more general phenomenon and is not unique to *S. marcescens*.

### Biofilm force generation and associated substrate deformations

The observed increase in biofilm edge velocity with substrate stiffness might be surprising given that biofilm expansion rates decrease on substrates of increasing agar concentration. Increasing agar concentration has the combined effect of increasing substrate stiffness, the viscous loss modulus G’’, and decreasing substrate permeability, which hinders the flow of nutrients to the biofilm. Thus, the effects of stiffness cannot be unambiguously related to colony expansion rates.

**Fig. 6.**
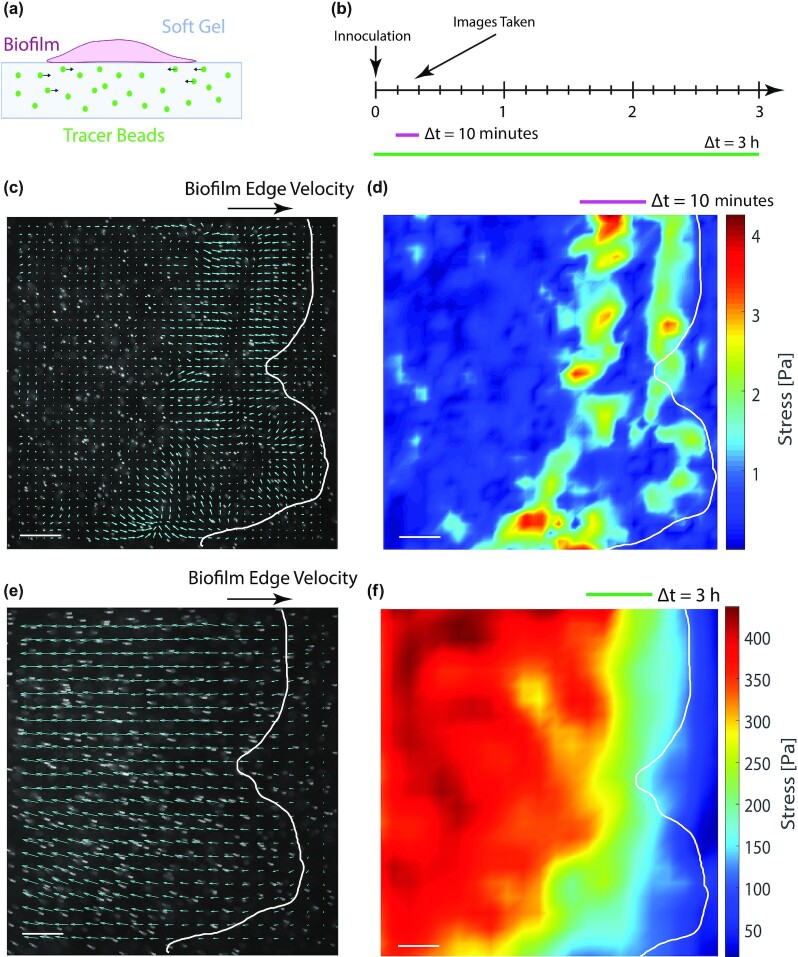
*Serratia marcescens* colonies exert collective time-dependent forces on their substrate. (a) Schematic of experimental set-up. Fluorescent tracers are embedded in the growth substrate underneath the colony. (b) Images are taken at 10-minute increments after inoculation up to 3 hours later. The displacement of the substrate is measured via particle imaging velocimetry (PIV; Methods), and the substrate displacement map depends on the time scale over which the video is observed (e.g. 10 minutes vs. 3 hours). (c) Over a relatively short time window (10 minutes) the displacement map and the (d) stress map show local regions of correlated motion near the expanding colony edge (overlaid in white). (e) Over longer time scales (3 hours), local fluctuations average out and there is a net drift of the particles toward the center of the colony, suggesting an inward contractile force. (f) The magnitude of the inner contractile force is much greater than the short-time fluctuations at the expanding edge. Data shown here is from a representative colony on a soft 0.5 kPa gel (3.5% PAA and 0.15% bis-crosslinker). Scale bar, 200 μm.

What may cause substrate elasticity to increase biofilm expansion rates on PAA gels? To better understand the observed enhancement in biofilm expansion with increasing substrate stiffness, we performed experiments in which biofilm-generated substrate displacements could be directly visualized via TFM-based techniques (Fig. 6). PAA deforms in proportion to applied forces and recovers completely and instantaneously on the release of the force. The displacements of PAA substrates are thus related to the stress at the surface, which can be reconstructed from the displacement fields based on the theory of linear elastostatics (42*–*44). However, if the substrate is nonlinear or viscoelastic (such as agar), then the relationship between stress and strain is much more complicated and time-dependent, and the substrate stress cannot be directly reconstructed from the substrate displacements.

**Fig. 7.**
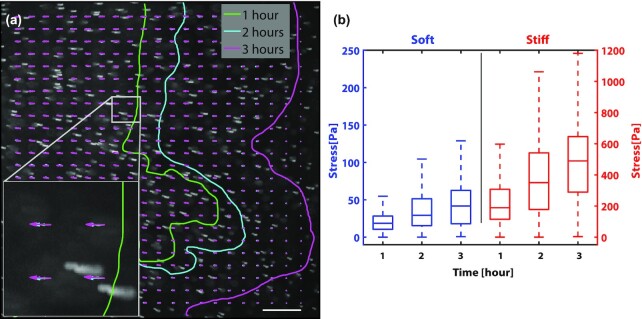
*Serratia marcescens* colonies generate more force on stiffer substrates. (a) A representative movie z-stack showing the trajectories of tracer particles embedded in the underlying substrate and in the vicinity of the expanding edge of the bacteria colony. Overlaid on the image are PIV displacement maps at 3 time points—1 hour (green), 2 hours (blue), and 3 hours (magenta)—as the colony grows over time. Inset shows increasing length of the displacement arrows, as the beads move further toward the center of the colony with each increasing hour. Also overlaid on the image are the colony boundary positions at hours 1, 2, and 3. Scale bar, 200 μm. (b) Box-and-whisker plots of the stress map points for hours 1, 2, and 3 for colonies growing on soft (0.5 kPa; 3.5% PAA, and 0.15% bis) and stiff (5 kPa; 8% PAA, and 0.15% bis) PAA gels. The stress grows over time and is significantly higher for colonies on stiffer substrates than soft ones. Data curated from 3 separate colonies per condition.

To determine whether substrate stiffness impacted the colony's ability to generate forces, we used TFM-based techniques to measure the stress exerted on the substrate by the expanding biofilms (Figs 6 and 7). To visualize the deformations of the substrate displacement, the PAA gels were embedded with 4.8 μm fluorescent beads, which were tracked over time. Using this technique, we observed two main types of substrate displacement. The first type occurred in the vicinity of the expanding edge of the colony as transient localized hot spots on the scale of 20 μm, much larger than an individual bacterium (Fig. 6c and d; Video S4). These localized regions were reminiscent of traction hotspots observed generated by collective motion *M. xanthus* cells (45). Here, these transient localized pulses were more evident on soft substrates (G’ = 0.5 kPa) for *S. marcescens* colonies than on stiff ones (G’ = 5 kPa). In addition to these hot spots, we observed a slower—but more consistent—inward motion of the beads toward the center of the colony (Figs 6d, f, and 7), consistent with the build-up of a bulk inward contractile force (26). In some of the TFM-based experiments, the fluorescent beads are applied only to the surface of the gel to more precisely track motion only at the surface of the gel. The fluorescent particles typically remained in focus throughout the entire experiment, suggesting minimal z-displacements. Assuming perfect focus at the start of the experiment, then the particles are displaced from center focus to half the depth of field, or 4 µm (0.5 × 8.5 µm) for the 10x objective used in these experiments, which is consistent with vertical substrate deformations on the order of 1–10 μm observed for *V. cholerae* and *P. aeruginosa* biofilms (46).

To estimate the forces exerted by the colony on the substrate, the stress at the surface was reconstructed from the tracer displacement maps via the finite element method (FEM) ( 47*–*49) (Methods). The stress at the surface increased over time, and—surprisingly—the stress was 10-fold higher for colonies on stiff substrates compared to soft ones (Fig. 7b): the average stress was approximately 500 Pa on the stiff gel compared to 40 Pa on the soft gel (3 hours time point). Typical surface strains $\epsilon $ also increased for biofilms on stiff substrates compared to soft ones, with surface strains at approximately 2% on stiff substrates compared to 0.5% on soft substrates (Methods). The large difference in bacteria force generation on soft vs. stiff substrates indicates a strong role of substrate stiffness on biofilm expansion and biofilm force generation.

To interpret these results (Figs 4, 6, and 7), we suggest a minimal model that treats substrate deformations as a signature of poroelastic stresses in the network driven by osmotic pressure gradients across the growing biofilm front. In this picture, the hydrogel substrate behaves as a soft network permeated by nutrient fluid that can move relative to this network. Biofilm growth proceeds as bacteria divide and begin to excrete extracellular polymers. Before these polymers assemble into the extracellular matrix network, they act as osmolytes that set up an osmotic pressure difference between the biofilm and the substrate (10, 16, 18). Gradients in osmotic pressure draw up fluid and nutrients into the biofilm, which allows the biofilm front to grow and expand. Osmotic spreading of biofilms was first observed for bacteria on agar substrates (10, 11). As seen on agar, decreasing network pore size reduces fluid permeability and diminishes colony expansion. Detailed 3D flows on agar have not been fully resolved; we also note that on viscoelastic substrates, such as agar, understanding the flows and stresses in the network are complicated by the nonlinear mechanics and viscous dissipation that alleviate stresses over time.

**Fig. 8.**
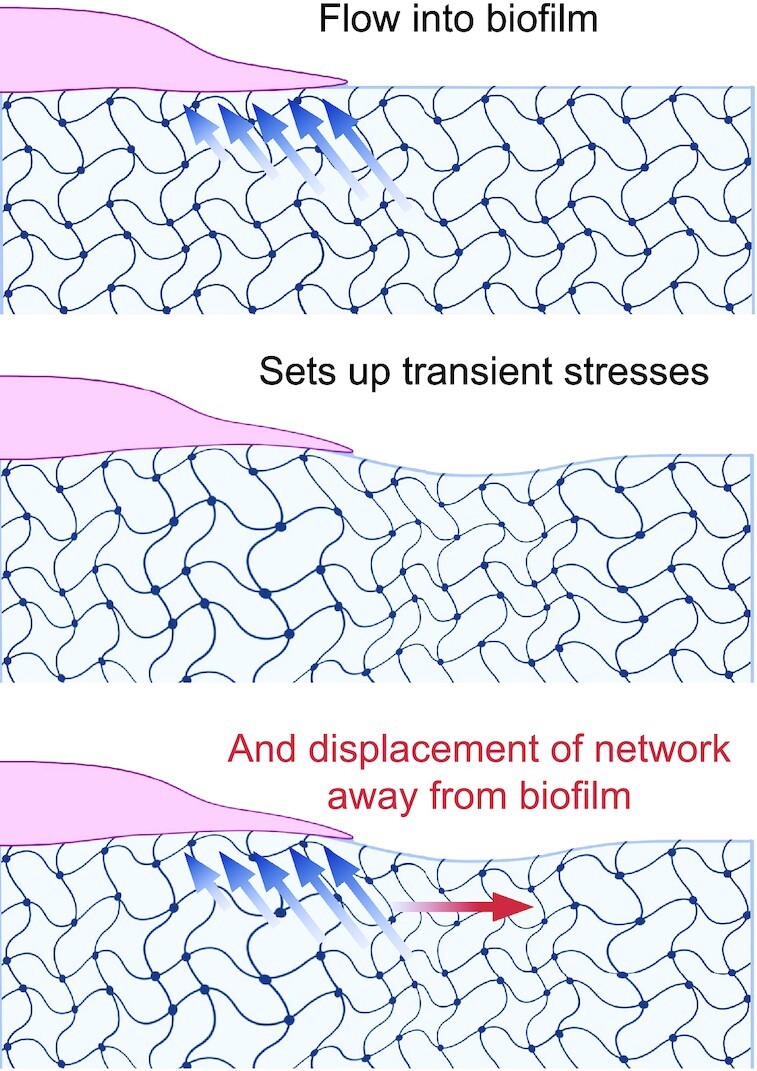
Schematic representation of poroelastic stresses associated with a growing biofilm front.

Here, we use PAA gels without viscous dissipation and tunable pore sizes to quantify biofilm expansion rates. Our results suggest that the larger stresses induced on stiffer substrates provides higher nutrient fluid flows that induce higher rates of biofilm growth. Motivated by our findings and the results of others (18, 46), we propose that this fluid flow sets up transient stresses in the substrate network, which could drive substrate displacements in regions surrounding the biofilm. In a poroelastic material, fluid flows and network deformations are coupled. This is shown schematically in Fig. 8, in which vertical indentations of the substrate are exaggerated to illustrate the effect of the colony osmotic pressure on the substrate. Based on our experimental observations (Figs 4 and 6), we infer that stiffer substrate networks more efficiently couple with fluid flows, increasing transmission of forces through the network and driving enhanced transport of the fluid through the network . On soft substrates, in contrast, local strains decay faster, resulting in reduced propagation and transmission of stress. The flow of fluid to relax the applied stress is, thus localized to smaller regions resulting in reduced fluid and nutrient flux. In this way, substrate network stiffness may act to increase initial biofilm growth rates. If the substrate is viscoelastic, such as agar, then viscous stress dissipation might further reduce flow. Taken together, our experiments highlight complementary roles played by fluid flows and network strength properties of substrates on which biofilms growth. For growing *S. marcescens* colonies, increased substrate stiffness enhances biofilm growth rates.

## Discussion

Bacteria are capable of transducing mechanical signals from their environment and responding to those cues (50*–*52), but the precise mechanisms remain largely unclear. In this article, we investigated the effects of substrate material properties on the biofilm expansion of *S. marcescens*. Using PAA hydrogels of varying composition, we found that substrate stiffness and porosity tune the spread of growing biofilm colonies. Our results indicated that increasing substrate stiffness enhances biofilm expansion rates in the limit of purely elastic substrates, unlike conventional agar substrates.

Taken together, our results suggest that substrate stiffness and substrate pore size have two different effects on colony expansion. Increasing pore size enhances biofilm expansion (Fig. 4). This result is largely expected as larger network pore sizes allow for enhanced diffusion and flow of nutrients from the substrate into the biofilm. An unexpected finding here is that substrate stiffness can have as big an impact on biofilm expansion rates as network pore size, and increasing substrate stiffness increases colony expansion (Fig. 4), even when network pore size is controlled and accounted for.

In the case of agar, substrate stiffness and pore size are coupled. Decreasing pore size and permeability of the network may be the limiting factor in growth, as the biofilms have limited access to nutrients. Another factor on agar substrates is its nontrivial mechanical properties (*53*). Agar has a viscous loss modulus that is as large as 50% its elastic storage modulus (Fig. 1). Thus, agar behaves as a viscoelastic solid and will dissipate applied stress over time scales of minutes relevant to biofilm growth. Since our data suggests that poroelastic stress in the network promotes fluid flows that deliver nutrients to the biofilm, the effect of viscous dissipation in agar substrates might further limit biofilm growth.

Our interpretation of colony surface expansion data makes a number of assumptions and simplifications. Here, we measure the expansion rate of the biofilm as an important metric of cooperative surface dispersal. We do not ascertain to which degree the expansion of the colony arises from increases in cell motility, cell division rates, EPS production, surfactants, or the amount of nutrient availability in the substrate. We do not measure specific genes transcribed during initial cell attachment that are required for biofilm differentiation (54). Biofilms are known to preferentially form under conditions of external fluid shear flows (55), where there may also be continual renewal of nutrients (56). Biofilms grown under shear conditions are known to express global gene expression profiles that differ from planktonic bacteria and colonies grown under agar (56); however, the production of EPS in our colonies (Figure S4, Supplementary Material) indicate some of the bacterial cells show some characteristics of biofilm growth.

An emerging number of studies indicate that bacteria sense surfaces by translating mechanical cues presented by the surrounding environment into biochemical signals through mechanosensitive signaling pathways (50, 52, 57). At the scale of an individual bacterium, there are now several molecular machines identified that can read-out mechanical signals, such as the bacteria flagella (58*–*60), pili (50, 57), and cell envelope ion-channels (61*–*63). These signals allow bacteria to modulate gene expression, cellular differentiation, and virulence factors (50) in response to physical changes in their environment. An advantage of PAA gels is that we can comprise gels of increasing stiffness with minimal changes in the surface network by modulating the cross-linker density instead of the monomeric acrylamide. It has been hypothesized that biofilm activation might be faster on stiffer substrates, because bacteria make contact with the substrate network through force-sensitive appendages, such as flagella or pili, at a higher frequency, increasing the possible input for cells to differentiate into a biofilm state. In contrast to the view, here, we find that enhanced surface dispersal of EPS-producing colonies occurs even under cases when the surface network is relatively unchanged (Fig. 4b).

Our results are consistent with a model of bacteria colony expansion driven by osmotic swelling (10, 11) and the poroelastic response of the underlying substrate. Here, we propose that in the context of bacteria colonies, one source of stress is due to osmotic pressure and pressure gradients that drive swelling and deswelling deformations of the gel substrate. Interestingly, swelling and deswelling deformations have also been recognized in traction force-based experiments in the epithelial cell sheet systems ( 64). Bacteria colonies are thought to exert different modalities of substrate stresses, including osmotic stress ( 10, 11), friction ( 21) and internal contractile forces (46). Our TFM based measurements represent a superposition of these effects; there is currently no obvious way to disentangle the stress from these different sources experimentally. One assumption in our traction force-based analysis is that the applied forces are tangential to the substrate surface. Three-dimensional substrate deformations can be assessed by computing the divergence of the deformation fields, as defined by $\nabla \cdot \overrightarrow{v} = \frac{\partial v_x}{\partial x} + \frac{\partial v_y}{\partial y}$. We computed the divergence of the substrate deformation fields (Fig. 6) which on average were negligible. Interestingly, a wave of non-zero substrate divergence (of unitless magnitude 0.015 over a displacement window of 5 minutes) was observed in some of our experiments, traveling along with the expanding edge of the growing colony. These results are consistent with localized transient osmotic pressures at the expanding colony edge (Fig. 6).

Here, we demonstrate that *S. marcescens* colonies are capable of responding to changes in substrate stiffness by modulating the amount of stress that they exert on their substrates, enhancing the applied stress with increasing substrate stiffness (Figs 6 and 7). One possible reason is an increased activation in biofilm formation genes that increases rates of EPS production, which form a filamentous network that is better able to transmit forces within the colony and to the surface. We also find a correlation between the colony substrate stress and colony expansion: colony expansion is faster when the colony stress is high. Both colony expansion rates and colony stress increase with substrate stress, but precisely how colony stress and substrate stiffness modulate expansion rates is not yet clear.

There are number of human infections involving biofilms (2, 54). Biofilms are implicated in cystic fibrosis, gingival disease, pneumonia, urinary tract infections, ear infections, and implant infections (2, 54). *Serratia marcescens* used in this study is an opportunistic bacterium implicated in a range of infections, including urinary and respiratory infections (65). While the genes required for biofilm formation have been extensively studied from the point of view of the microbe, there is much less-known regarding the requirements for bacteria to infect the soft tissue of their host. A number of recent studies have illuminated the role of substrate stiffness on cell attachment (19, 20) and growth and have demonstrated that bacteria can exert direct forces that remodel and disrupt host tissue (46). Human tissues that bacteria infect vary in shear stiffness, ranging from 10 to 100 Pa for mucus and 10 kPa for lung to 100–1,000 kPa for skin and gut (66*–*68). Inflammation and disease can further alter host tissue stiffness (68*–*70). Our work here shows that the mechanical properties of extracellular environment impact colony expansion, which has important implications for understanding the infection of soft tissues in vivo.

Our results provide compelling evidence that biofilms can respond to the mechanical properties of their environment beyond single cells and at the collective cell level. Our results suggest new models of biofilm growth that explicitly account for the effects of substrate stiffness and poroelastic substrate remodeling. Much more work is needed of course, and in this regard we note that the PAA gels presented here can be adapted to investigate the effect of specific adhesion factor presented on the surface or to systemically introduce substrate viscoelasticity (25, 71). PAA hydrogels offer a conceptually simple platform for studying how substrate stiffness impacts bacteria surface dispersal and guiding our understanding of collective colony growth.

## Methods

### Cell culture

There were 4 strains of bacteria used in this study*: S. marcescens* (274 ATCC), *P. aeruginosa* (Xen05), *P. Mirabilis* (BB2000), and *M. xanthus* (DK1622). With the exception of *M. xanthus*, bacteria cells were inoculated and grown in LB medium with shaking at 37°C overnight. *Myxococcus xanthus* was inoculated and grown in CTTYE medium. The cell density was measured at OD600 using 1-cm cuvettes (Globe Scientific 112137) and a spectrophotometer (Thermo Fisher Scientific Genesys 50). Cell suspensions were then diluted to 0.6 at OD600 in cell medium. For all bacterial strains, 5 μL of inoculum was spotted on growth substrates (agar or PAA gels of varying stiffness). Cultures were then maintained at 37°C (or 30°C for *M. xanthus*) for up to 15 hours. *Pseudomonas aeruginosa* Xen05 was kindly provided by Dr Robert Bucki (Medical University of Białystok), and *P. Mirabilis* (BB2000) by Dr Karine Gibbs (Harvard University).

### Gel preparation

To prepare hydrogels of varying stiffness, PAA gels were prepared as described previously (72, 73). Briefly, PAA gels were prepared by mixing together acrylamide, bis-acrylamide, and distilled water at various ratios. Polymerization was initiated by the addition of 0.5 μL electrophoresis grade tetramethylethylenediamine (TEMED) followed by 1.5 μL of 2% ammonium per-sulfate (APS) per 200 μL of final gel solution. A total of 200 μL of the solution were then pipetted between two glass coverslips, one treated with glutaraldehyde (bottom) and the other SurfaSil-treated (top), and allowed to polymerize for 20 minutes. Then, the top cover slip was removed from the gels, and the final dimensions of the hydrogel formed a disc, 18 mm in diameter and 0.8 mm in height. For TFM experiments, fluorescent beads (4.8 μm diameter Fluroro-Max polymer microspheres) were embedded in the gels by using a 1:20 dilution of the bead solution in distilled water. The dilution was performed after centrifuging the bead solution and replacing the supernatant surfactant with distilled water. For a complete list of the gel formulations used in this manuscript, please see Table S1 (Supplementary Material).

### Rheological characterization

Rheology measurements were performed on a Malvern Panalytical Kinexus Ultra + rheometer equipped with a 20 mm diameter plate. The elastic gel solutions were polymerized at room temperatures between the rheometer plates at a gap height of 1 mm (30 minutes). The shear modulus was then measured as a function of shear strain from 2% to 50% at a frequency of 1 radian/second. For agar, G’ was chosen as the shear stiffness in the limit of 0% shear strain.

### Substrate preparation and inoculation

To prepare PAA substrates for inoculation, we followed a protocol previously described by Tuson et al (74). The PAA gels were washed 3 times (two 10-minute washes and one overnight wash) in phosphate-buffered saline (or TPM buffer for *M. xanthus*). The washes were then repeated with LB medium (or CTTYE medium for *M. xanthus*). Before inoculation, the substrates were removed from growth medium, allowed to dry for 20 minutes at room temperature, and then treated with UV sterilization for an additional 20 minutes. The prepared bacterial solution was inoculated onto the center of each gel in a 5 μL droplet. After placing the droplets, 2 μL of liquid was removed from each droplet with a pipette to bring bacteria in closer contact with the gel surface.

### Imaging

Time-lapse imaging was performed with a Nikon Ti-E inverted microscope equipped with a 4x objective. The cultures were maintained at 37°C (or 30°C for *M. xanthus*) using a Tokai-Hit stage top incubator. Images were taken every 10 minutes for 15 hours using a motorized stage to capture growth at 4 positions along the edge of each biofilm. After the 15 hours had elapsed, full colony images were taken with a MotiCam camera or using NIS Elements software to automatically stitch together multiple images taken with a 2x objective.

### Motility measurements

Time-lapse images were loaded in custom Python scripts that allowed manual supervision of automated boundary detection (Video S1). The boundaries were fit to circular arcs, and the average length of multiple radial lines connecting subsequent arcs determined the biofilm velocity. (Fig. 2b) Velocities are measured over 20-minute time increments. The colony expansion rate was measured at 4 different imaging windows along the periphery of each colony, and the mean expansion rate was computed for each colony. Velocities were measured at the earliest time at which expansion was present across gel conditions, which varied by species. For the data reported in Fig. 4 (*S. marcescens*), velocities were measured after 2 hours of growth. For the data reported in Fig. 5, velocities were measured at 6 hours for *P. aeruginosa*, 3 hours for *P. mirabilis*, and 10 hours for *M. xanthus*. Colony expansion rate data presented in Figs 4 and 5 are computed from the mean of 3–6 independent bacteria colonies per condition. Each experiment condition was verified from at least 2 separate inoculations of the bacterial stock on different days. Error bars denote the SEM.

### TFM-based methods

For TFM, bacteria were placed on PAA gels with embedded 4.8 μm fluorescent beads. Deformation of the PAA gel was captured by time-lapse imaging of the fluorescent beads during biofilm growth. The displacement field on the PAA gel generated by the bacteria was calculated by correlating time-lapse fluorescence images relative to the first frame of the sequence with particle imaging velocimetry (PIV) (75). The displacement field is then corrected for stage drift by subtracting the displacement field generated from the fluorescent beads’ images of the stress-free region of the PAA gel (far from the biofilm). The stresses that the biofilm exerts on the substrate can then be reconstructed from this deformation field using the FEM (47*–*49). In brief, the gel was modeled as a 3D block with a thickness of 1 mm. The biofilm and PAA gel were meshed with 4-noded tetrahedral 3D solid elements using a meshing algorithm. Forces with the same magnitude but opposing direction to the local stresses were applied to each node. Internal strains and stresses were then computed based on the geometry and elastic properties of the gel. The stress calculated is measured relative to the (prestressed) first frame of the imaging sequence (accumulated stress). The computation routine was performed using MATLAB and ANSYS Mechanical APDL. For instantaneous stresses, the displacement field is generated by comparing 2 consecutive frames of the captured fluorescent beads images. Subsequent analysis follows the same protocol described for accumulated stress above. Surface shear strain $\epsilon $ was estimated using the relation $\tau \ = \ G\epsilon $, where $\tau $ is the stress at the surface given by TFM and $G$ is the shear modulus measured from rheology.

## Supplementary Material

pgac025_Supplemental_Files

## Data Availability

This manuscript includes new custom Python code and new experimental data. The Python scripts for supervised tracing of biofilm images and the rest of the analysis pipeline are currently available at https://github.com/masp01/SUBII-Trace, along with all data presented in figures, and a summary of all experiments that were conducted. The results from this manuscript are gathered from video data of growing biofilms. We have analyzed over 15 experimental conditions, with 8+ videos per condition. In total, this is approximately 1 TB of data. Upon request, any of the raw images can be made available by contacting aepatteson@syr.edu.
