# Supplementary material for: Spreading rates of bacterial colonies depend on substrate stiffness and permeability
Source: PNAS Nexus. 2022 Apr 15;1(1):pgac025. doi: 10.1093/pnasnexus/pgac025 (PMC9802340; doi:10.1093/pnasnexus/pgac025)
Supplement: pgac025_Supplemental_Files [file pgac025_supplemental_files.zip › PNASNEXUS-PNASNEXUS-2021-00185-s05.pdf]

Data Management

This manuscript includes new custom Python code and new experimental data.

The Python scripts for supervised tracing of biofilm images and the rest of the analysis pipeline are currently available at https://github.com/masp01/SUBII-Trace, along with all data presented in figures, and a summary of all experiments that were conducted.

The results from this manuscript are gathered from video data of growing biofilms~~.~~ We have analyzed over 15 experimental conditions, with 8+ videos per condition. In total, this is approximately 1 TB of data.

Upon request, any of the raw images can be made available by contacting aepatteson@syr.edu.

GitHub

GitHub - masp01/SUBII-Trace
