# Supplementary material for: Spreading rates of bacterial colonies depend on substrate stiffness and permeability
Source: PNAS Nexus. 2022 Apr 15;1(1):pgac025. doi: 10.1093/pnasnexus/pgac025 (PMC9802340; doi:10.1093/pnasnexus/pgac025)
Supplement: pgac025_Supplemental_Files [file pgac025_supplemental_files.zip › Supplementary-information-corrected.pdf]

Supplementary information for “Spreading rates of bacterial colonies depend on substrate stiffness and permeability”

Merrill E. Asp[1,2], Minh Tri Ho Thanh[1,2], Danielle A. Germann[1,2], Robert J. Carroll[1,2], Alana Franceski[2,3], Roy D. Welch[2,3], Arvind Gopinath[4,5] and Alison E. Patteson*[1,2]

1 Physics Department, Syracuse University

2 BioInspired Institute, Syracuse University

3 Biology Department, Syracuse University

4 Department of Bioengineering, University of California, Merced

5 Health Sciences Research Institute, University of California, Merced

% PAA	% Bis	G’ (kPa)
3	0.15	0.14 ± 0.02
3.5	0.15	(0.5)
4	0.15	0.94 ± 0.09
6	0.15	2.72 ± 0.13
8	0.15	5.34 ± 0.35
10	0.15	(10)
12	0.15	12.0 ± 0.93
8	0.02	0.68 ± 0.14
8	0.05	1.62 ± 0.31
8	0.085	(3.5)
8	0.2	6.02 ± 0.59
8	0.3	8.82 ± 1.09
8	0.45	(10)
8	0.6	11.8 ± 0.42

Table S1. Summary of hydrogel compositions and their corresponding storage modulus *G’*. Reported values are mean ± standard deviation, as measured by Malvern Panalytical Kinexus Ultra+ stress-controlled rheometer (details in Methods section). Values in parentheses are estimated based on linear fits between experimental points from Figure 2c and 2d as appropriate.

% PAA	% Bis	Contact Angle
4	0.15	15° ± 2°
8	0.15	23.5° ± 1°

n ≥ 3 measurements

Table S2. Summary of contact angle (mean ± standard deviation) of water on the surface of PAA hydrogels. These measurements were made using a contact angle goniometer.

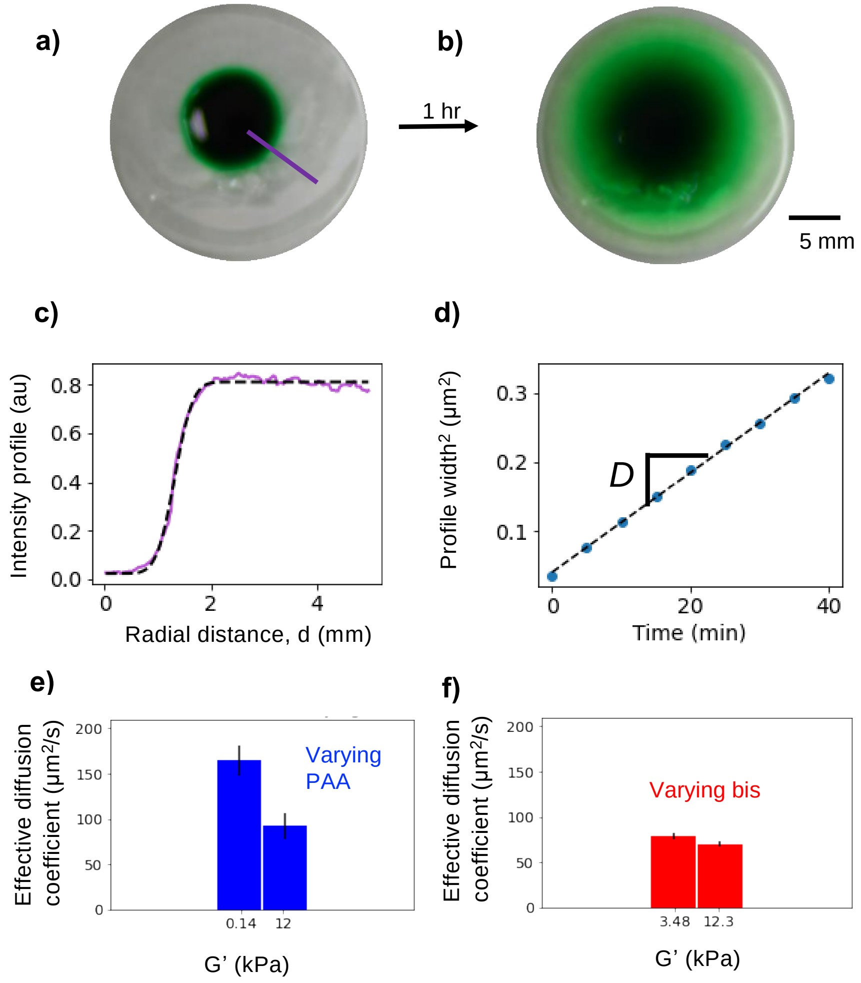

Figure S1. Characterizing effective transport of a dye through polyacrylamide gels. (a&b) A drop of green food-coloring dye spreads through a PAA gel, pictured at **(a)** initial and **(b)** final (60 min) time points. The transport of the dye is due to fluxes arising as the gel changes state and swells or de-swells as well as due to molecular transport. **(c)** The intensity profile of the diffusing dye is fit to an error function with a specific width *w* (initial profile shown). **(d)** The slope of w^2 over time gives the effective (transport) diffusion constant *D* for the hydrogel. **(e&f)** The effective diffusion constant for gels with varying **(e)** PAA concentration versus varying **(f)** bis concentration. Results show that *D* decreases with increasing PAA concentration, but is approximately constant for gels with increasing bis concentration. These data span the stiffness range used in this work. The formulation for the gels shown in (e) and (f) are in order from left to right: 3% PAA, 0.15% Bis; 12% PAA, 0.15% Bis; 0.085%, 8% PAA; and 0.45% Bis, 8% PAA.

Quantifying matrix permeability

To quantify the permeability of the gel networks, we used a simple dye diffusion experiment (Fig. S1). Drops of green-food coloring dye were placed on the surface of the gels and allowed to disperse through the gel over time. The evolution of the dye was tracked via images taken 10 minutes apart (Fig. S1a&b). Here, the image intensity correlates with the concentration of the

dye. The intensity profile across the expanding edge of the droplet was well-approximated by an error function. Thus, we fit the intensity profile curves to an error function to obtain a profile width *w* over time (Fig. S1c&d). The width of the intensity profile increased over time and the effective diffusion *D* of the dye through the gel could be estimated by the slope of the w^2 over time (Fig. S1d). The diffusivity values measured in our gels varies from approximately 50 to 150 $\mu m^2/s$.

Here, the diffusion coefficient that we measure is an effective dispersion coefficient, which combines these effects of molecular diffusion and characterizes the transport of solvent as the gel swells. Our results suggest that a significant component of the transport is due to fluxes arising from a redistribution of fluid in the gel as it swells.

Estimating effective matrix pore size

Our results suggest that the development of biofilms on soft, porous substrates depends on both the elasticity of the gel and its permeability. The permeability of the substrate controls the resistance to the flow of nutrient fluxes through the gel matrix. Next, we combined rheological data and diffusion experiments to compute a network permeability *k* and estimate an effective pore size r_p of the networks relevant to osmotic-induced spreading of bacteria colonies.

Here, we assume that the polyacrylamide substrates act as poroelastic gels that can swell. Poroelastic relaxations are associated with fluxes that aim to re-establish chemically equilibrated states occur in a characteristic time τ_p. During the deformation or stressing of highly swollen polymer networks, mechanical deformation is coupled to the mass transport of solvent through the network. Localized stresses such as those exerted by a large area of the biofilm results in substrate deformation and causes a chemical potential gradient to develop within the hydrogel close to the interface. Over time, the hydrogel establishes a new chemical equilibrium via migration of liquid (here the nutrient solution) away or from the region under deformation. Due to this process, the hydrogel undergoes a load relaxation that reflects the time-scale to establish this new equilibrium τ_p. The volume of the deformed region establishes the volume of the nutrient that must migrate, the relaxation process depends on the area over which the gel is deformed or chemical stressed are induced

To estimate the permeability *k* of a gel, we first note the equation relating the (poroelastic) diffusivity D to the shear modulus G, the Poisson ratio ν, the permeability k, and the medium viscosity η,

$$D = \frac{2(1-\nu)}{(1-2\nu)} \frac{Gk}{\eta} .$$

The diffusivity provides the length scale $\sqrt{Dt}$ over which nutrient concentration is transported due to fluid fluxes (local induced pressure gradients) in a time t due to imposed deformations. The Poisson ratio ν characterizes the ability of the gel to swell. The permeability, Poisson ratio and the shear modulus depend on the degree of swelling of the gel.

The effective poroelastic diffusion coefficients for different PAA substrates D, are roughly estimated by analyzing the radial spread of a small molecule dye (Fig. S1) that can be transported by and move with fluid in the presence of fluxes, and the shear modulus *G* is measured via oscillatory tests (Fig. 1 main text). Previous studies reported an estimated

Poisson ratio of around 0.25-0.4 for PAA gels with concentrations of 5 wt% monomer when swollen significantly, which we choose to measure here a compression experiment using the rheometer (Fig. S2). Knowing $D, G, \upsilon,$ and using the viscosity of the solvent permeating the gel (approximating it to be that of water), we can compute the permeability k.

To calculate the Poisson ratio, we use a parallel-plate rheometer to apply a small uniaxial compression. The gel thickness, H = 1.0 mm, is compressed by a vertical amount $\delta \ll H$. The top plate of the rheometer is a flat disc of radius R and the volume of the indented region is $\pi R^2 \delta$. The normal force on the top plate from the sample is measured over time (Fig. S3). Here the gel is unjacketed and kept in a medium that allows for motion of fluid through the outer boundaries.

Upon compression, the normal force exhibits a steep instantaneous rise in value, which subsequently relaxes over time. At short times, the gel behaves as an incompressible material since the fluid does not have time to flow out of the region – this provides an instantaneous load F_0 that eventually relaxes to a long-time limiting value F_∞. Ignoring the effects of tortuosity and assuming that the final state of indentation allows the gel to relax to a Poisson ratio that is its equilibrium value, we use the relationships [1–3]

$$\frac{F_0}{F_\infty} \approx 2(1 - \nu)$$

and

$$r_p \approx 2\left(\frac{\eta}{2}\frac{D(1-2\nu)}{G(1-\nu)}\right)^{\frac{1}{2}}.$$

Table S3 summarizes the elastic and transport coefficients measurements from this work. Our estimated Poisson ratios are in the range of 0.43 to 0.49 (Table S3). Assuming the viscosity of the fluid permeating the gel is close to that of water and ignoring the effects of tortuosity within the gel, the estimated mean pore size is in the range of 0.8 to 22 nm (Table S3), consistent with prior measurements. Note that the shear modulus does depend on the confinement and the compression. For small compressions, we may approximate the shear moduli by the uncompressed values.

% PAA	% Bis	G' (kPa)	Poisson Ratio	Pore size (nm)
3	0.15	0.14 ± 0.02	0.43 ± 0.06	22 ± 5.6
12	0.15	12.0 ± 0.93	0.49 ± 0.01	0.85 ± 0.14
8	0.085	(3.5)	0.49 ± 0.01	1.5 ± 0.11
8	0.45	(10)	0.49 ± 0.01	0.9 ± 0.07

Table S3. Parameters used to estimate effective pore size.

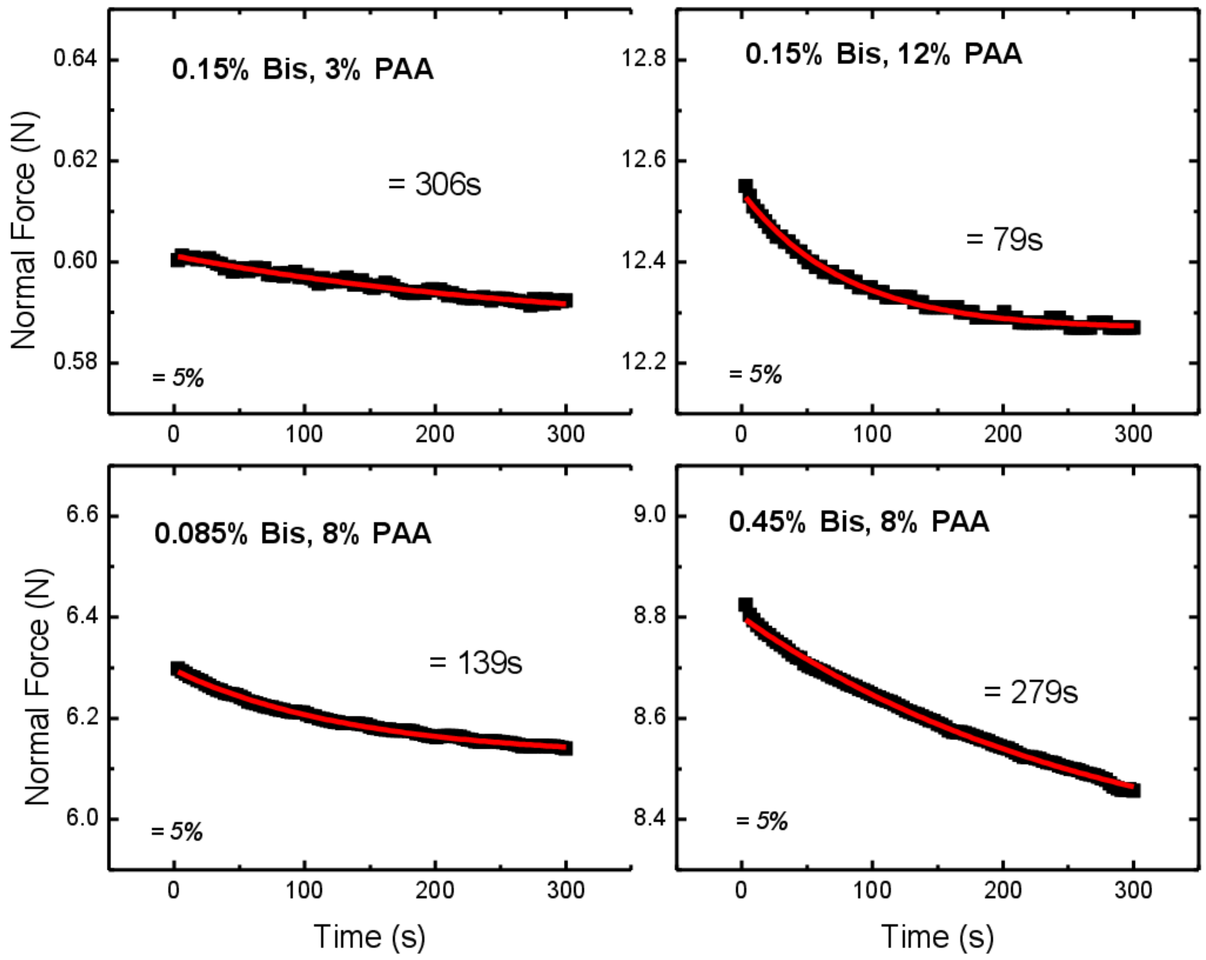

Figure S2. Normal force relaxation of PAA gels. Representative normal force decay curves from uniaxial compression of different PAA gel compositions. 5% uniaxial compression was applied to each gel and the normal force decay was measured with a parallel plate rheometer equipped with a 20 mm plate. The Poisson ratio ν was then given by $\frac{F_0}{F_\infty} \approx 2(1-\nu)$, where F_0 is the initial jump in force when the compression is applied and F_∞ is the final value of the force after three minutes of observation. We conducted three independent trials per condition to compute a mean and standard deviation.

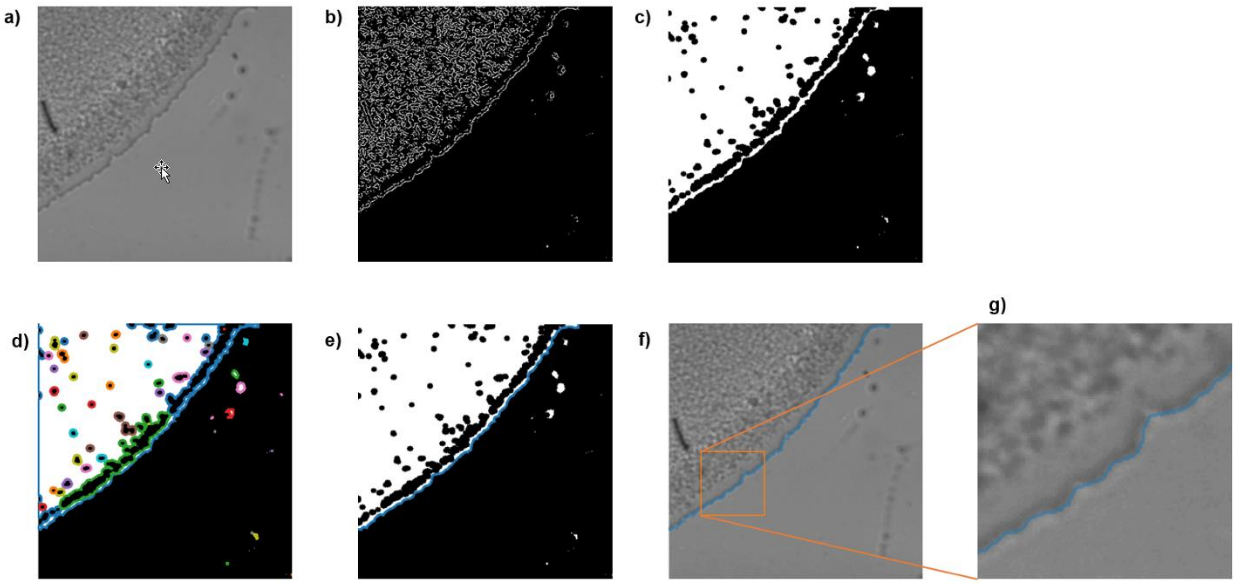

Figure S3. Automated biofilm boundary detection algorithm. (a) An unprocessed biofilm image. **(b)** Canny edge detection produces a binary image. **(c)** A circular kernel nine pixels wide smooths the boundaries and closes broken lines. **(d)** The function cv2.findContours identifies the boundaries around contiguous regions, each shown here in a different color. The segment with the longest edge-to-edge distance matches the biofilm boundary but often includes erroneous features around the finite edges of the image. **(e)** Since the boundary consistently intersects the edge of the picture, these erroneous features are easily removed by cutting the biofilm boundary segment at the picture edges. **(f)** The coordinates identified with this method follow the biofilm boundary with high precision. **(g)** Zoomed-in snapshot of boundary from (f).

Supervised biofilm boundary detection

The quantitative metric of biofilm growth used in this study is biofilm boundary velocity. To calculate the boundary velocity from a sequence of time lapse images, image processing is used first to automatically detect the coordinates of the biofilm boundary for each image. Then the detected boundary is manually verified and, if necessary, corrected before being further processed as explained in the Methods section.

The automatic boundary detection and manual correction steps were integrated into a single Python script to increase efficiency and ease of use. Highly detailed edge boundary coordinates can be extracted from many images in this way.

The Python implementation of the OpenCV library is used to implement the steps of the automatic boundary detection algorithm. Using the Anaconda distribution of Python, OpenCV can be installed as `pip install opencv-contrib-python`, and then used in scripts with `import cv2`. Loading images is accomplished with the PIMS package.

The automatic boundary detection algorithm consists of the following steps:

1. **Canny edge detection (`cv2.Canny`)**
   This produces a binary image where edges are shown as white pixels, and the rest of the image is black. Two parameters, a minimum and maximum gradient threshold are the only inputs.
2. **Morphological closing (`cv2.morphologyEx`)**
   This step closes the broken lines often produced by Canny edge detection by first dilating white pixels (i.e. outlining white pixels in white by a specific thickness, or kernel size), which bridges the gaps between nearby regions, and then eroding the white pixels using the same kernel size to obtain the same edge thickness as before.
3. **Closed contour identification (`cv2.findContours`)**
   This function identifies all the contiguous white regions in the image and returns the contour boundary positions for each one. The contour with the longest edge-to-edge distance consistently matches with the biofilm boundary (Fig. S3e).
4. **Contour segmentation**
   This step removes the portions of the closed contour that lie on the image edge, removing erroneous edge features and leaving line segments that extend from edge to edge of the image (Fig. S3e).
5. **Segment selection**
   Finally, the segment with the longest end-to-end distance is displayed as the predicted biofilm boundary.

Occasionally, an imperfection in the gel surface can get identified as part of the biofilm boundary. To address this issue, we developed a GUI that allows the user to click on a point on the automatically identified boundary and manually draw a new segment portion that ends on the boundary or the image edge. These manually drawn points replace the erroneously identified points, and the coordinates of the resulting biofilm boundary are saved by the user when all corrections are complete. A demo is shown in Video S1.

Taken together, this semi-automated boundary tracking code significantly reduces time spent in analysis. A typical experimental trial generates 1,000-2,000 images (12 individual colonies in a multi-well plate x 4 locations per colony x 3 frames/hr x 10 hours). Manual tracing of this many boundaries, in ImageJ with a digital pad for example, would require multiple days to complete. Using this code, 1000 individual images can be analyzed and verified for boundary identification in about one hour. The full code is available via Github at https://github.com/masp01/SUBII-Trace, along with sample biofilm images. Any data used in this study is available upon request.

The resolution of this technique depends on the closing kernel radius (step 2). Here, we used 4.5 pixels, or 14.5 µm with our 4x objective, which is larger than an individual cell but much smaller than the length of boundaries (several mm).

Biofilm matrix fluorescent imaging

To determine whether the bacterial colonies are excreting EPS, after 15 to 24 hours of growth, *Serratia marcescens* colonies were stained with Invitrogen Film Tracer SYPRO Ruby Biofilm Matrix Stain (Thermo Fisher F10318) according to the manufacturer's instructions. Fig. S4 shows a representative image of a biofilm with positive matrix staining. Adding the matrix strain slightly disrupts the colony structure, resulting in a diluted mixture as seen in the image.

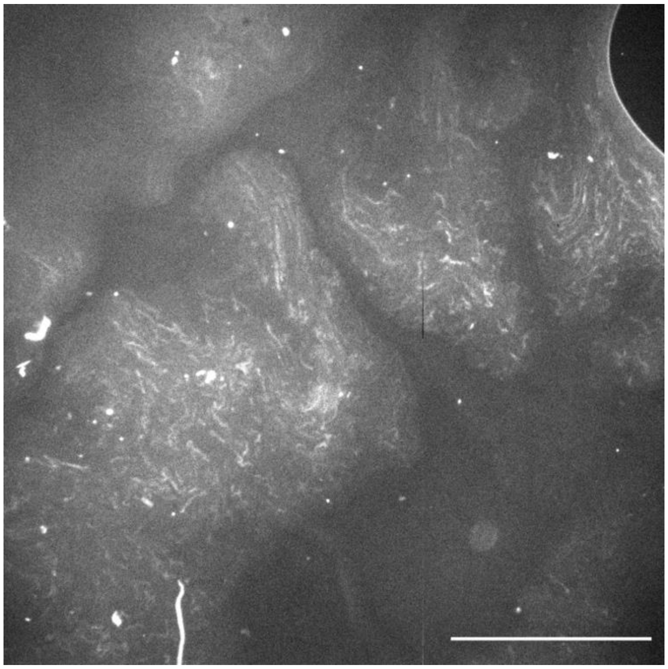

Figure S4. EPS fluorescent staining indicating biofilm development. The edge of the *Serratia marcescens* colony after 15 hours of growth is present in the upper-left corner. Biofilm matrix stain was applied to the surface of the biofilm, revealing regions of concentrated EPS that indicate biofilm development. Scale bar is 1 mm.

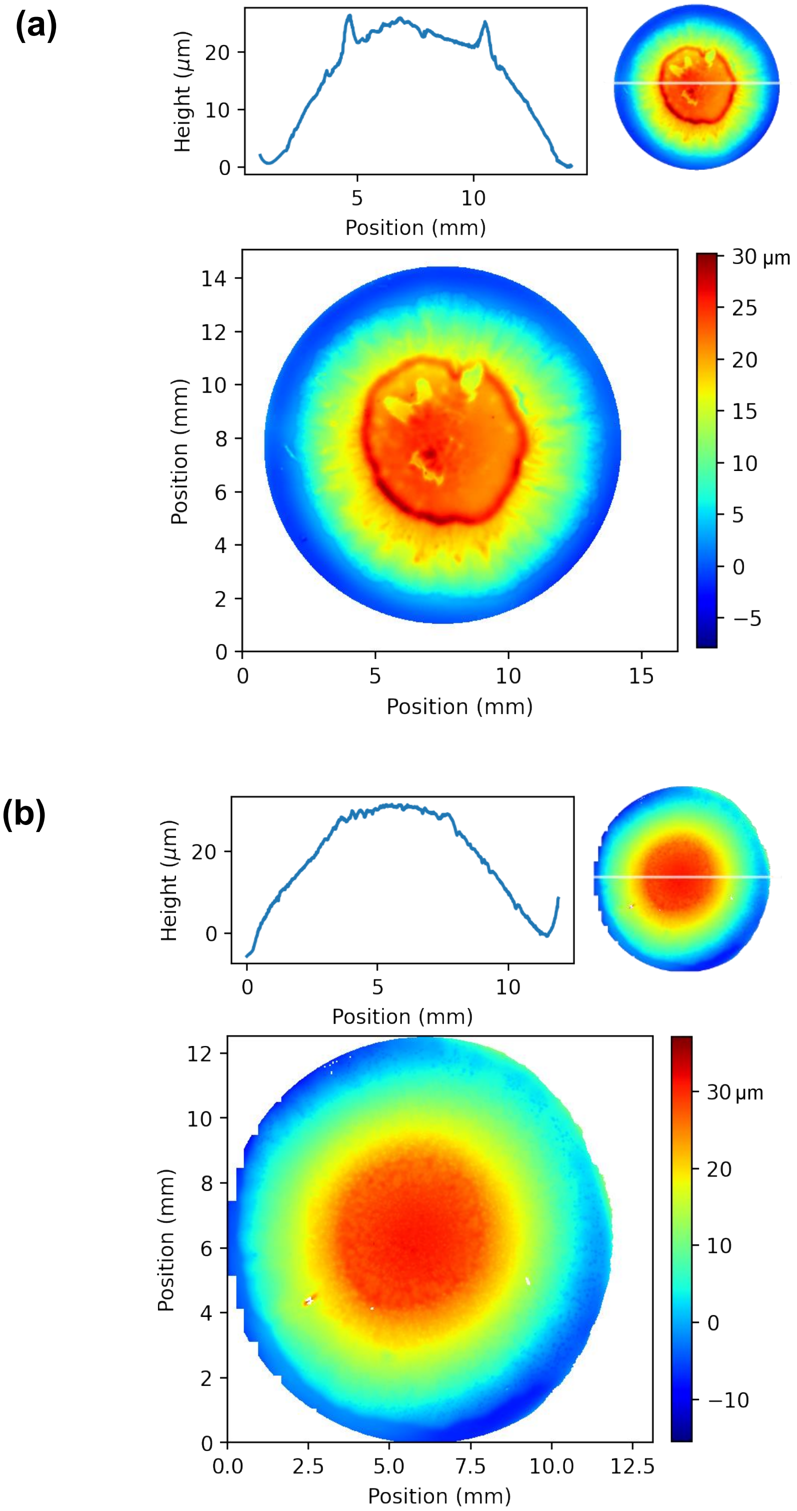

Figure S5. Three-dimensional landscape of bacteria colonies. The 3D colony landscape was mapped with a white light interferometer (Bruker CONTOURX-200). Representative interferometer images for *Serratia marcescens* colonies on: **(a)** a soft, 500 Pa gel (3.5% PAA, 0.15% Bis) and **(b)** a stiff, 5 kPa gel (8% PAA, 0.15% Bis).

References

[1] W. C. Lin, K. R. Shull, C. Y. Hui, and Y. Y. Lin, *Contact Measurement of Internal Fluid Flow within Poly(n- Isopropylacrylamide) Gels*, J. Chem. Phys. **127**, (2007).

[2] E. P. Chan, Y. Hu, P. M. Johnson, Z. Suo, and C. M. Stafford, *Spherical Indentation Testing of Poroelastic Relaxations in Thin Hydrogel Layers*, Soft Matter **8**, 1492 (2012).

[3] C. Y. Hui, Y. Y. Lin, F. U. C. Chuang, K. R. Shull, and W. C. Lin, *A Contact Mechanics Method for Characterizing the Elastic Properties and Permeability of Gels*, J. Polym. Sci. Part B Polym. Phys. **44**, 359 (2006).

Supplementary Video Captions

Video S1: Demonstration of the GUI that implements the automated boundary detection algorithm, allowing the user to quickly verify the boundary quality and make manual updates.

Video S2: Representative *Serratia marcescens* biofilm growing on a soft polyacrylamide hydrogel (G' = 0.9 kPa). This video was taken with a 4X objective at 10 minutes/frame for 15 hours. Playback is at 4200 x real-time speed.

Video S3: Representative *Serratia marcescens* biofilm growing on a stiff polyacrylamide hydrogel (G' = 3 kPa). This video was taken with a 4X objective at 10 minutes/frame for 15 hours. Playback is at 4200 x real-time speed.

Video S4: A *Serratia marcescens* biofilm growing on a soft polyacrylamide hydrogel (G’ = 0.9 kPa) with 4.8 µm diameter fluorescent beads embedded in the gel to display substrate displacements. During the first loop, the biofilm is shown in brightfield. During the second loop, only the fluorescent beads are shown. During the third loop, an outline indicating the biofilm boundary is overlaid. This video was taken with a 10X objective at 10 minutes/frame for approximately 1.5 hr. Playback is at 4200 x real-time speed.
